# Pancreatic and intestinal endocrine cells in zebrafish share common transcriptomic signatures and regulatory programmes

**DOI:** 10.1186/s12915-020-00840-1

**Published:** 2020-08-31

**Authors:** Arnaud Lavergne, Estefania Tarifeño-Saldivia, Justine Pirson, Anne-Sophie Reuter, Lydie Flasse, Isabelle Manfroid, Marianne L. Voz, Bernard Peers

**Affiliations:** 1 Laboratory of Zebrafish Development and Disease Models (ZDDM), GIGA, University of Liège, Avenue de l’Hôpital 1, B34, Sart Tilman, 4000 Liège, Belgium; 2 Present Address: Gene Expression and Regulation Laboratory, Department of Biochemistry and Molecular Biology, University of Concepción, Concepción, Chile

**Keywords:** Pancreatic endocrine cells, Enteroendocrine, Transcriptome, RNA-seq, Pax6, Zebrafish, Hormone, Transcriptome comparison

## Abstract

**Background:**

Endocrine cells of the zebrafish digestive system play an important role in regulating metabolism and include pancreatic endocrine cells (PECs) clustered in the islets of Langerhans and the enteroendocrine cells (EECs) scattered in the intestinal epithelium. Despite EECs and PECs are being located in distinct organs, their differentiation involves shared molecular mechanisms and transcription factors. However, their degree of relatedness remains unexplored. In this study, we investigated comprehensively the similarity of EECs and PECs by defining their transcriptomic landscape and comparing the regulatory programmes controlled by Pax6b, a key player in both EEC and PEC differentiations.

**Results:**

RNA sequencing was performed on EECs and PECs isolated from wild-type and *pax6b* mutant zebrafish. Data mining of wild-type zebrafish EEC data confirmed the expression of orthologues for most known mammalian EEC hormones, but also revealed the expression of three additional neuropeptide hormones (Proenkephalin-a, Calcitonin-a and Adcyap1a) not previously reported to be expressed by EECs in any species. Comparison of transcriptomes from EECs, PECs and other zebrafish tissues highlights a very close similarity between EECs and PECs, with more than 70% of genes being expressed in both endocrine cell types. Comparison of Pax6b-regulated genes in EECs and PECs revealed a significant overlap. *pax6b* loss-of-function does not affect the total number of EECs and PECs but instead disrupts the balance between endocrine cell subtypes, leading to an increase of ghrelin- and motilin-like-expressing cells in both the intestine and pancreas at the expense of other endocrine cells such as beta and delta cells in the pancreas and *pyyb*-expressing cells in the intestine. Finally, we show that the homeodomain of Pax6b is dispensable for its action in both EECs and PECs.

**Conclusion:**

We have analysed the transcriptomic landscape of wild-type and *pax6b* mutant zebrafish EECs and PECs. Our study highlights the close relatedness of EECs and PECs at the transcriptomic and regulatory levels, supporting the hypothesis of a common phylogenetic origin and underscoring the potential implication of EECs in metabolic diseases such as type 2 diabetes.

## Background

The enteroendocrine cells (EECs) and pancreatic endocrine cells (PECs) play a crucial role in the control of metabolism of all vertebrates. The PECs, notably via the antagonistic action of glucagon and insulin, secreted respectively by the alpha and beta endocrine cells, regulate glycaemia. The EECs also participate in this control through the release of some hormones, like glucagon-like peptide-1 (GLP-1) or glucose-dependent insulinotropic polypeptide (GIP), which display incretin effect, enhancing the glucose-stimulated insulin secretion. The EECs secrete more than 15 hormones that modulate many physiological processes including the release of digestive enzymes and bile from the exocrine pancreas and gallbladder, control of intestinal motility, sensing of nutrients and microbial metabolites and stimulation or suppression of appetite [1]. The EECs were initially classified into distinct subtypes according to the hormone they secrete; however, subsequent analyses revealed that there is an extensive co-expression of several hormones in individual EEC [2]. Recent single-cell RNA-seq studies have indeed confirmed this co-expression in murine EECs thereby identifying at least 9 EEC subtypes expressing various combinations of neuropeptides or hormones [3, 4].

EECs display similarities with pancreatic endocrine cells (PECs). Some hormones are produced from both pancreatic and intestinal endocrine cells, like somatostatin (Sst), ghrelin (Grhl) or peptide YY (Pyy) [5, 6]. Also, many transcription factors are involved in the differentiation of both EECs and PECs such as NeuroD, Pax6, Isl1, Nkx2.2, Insm1 and Arx [7–10]. For example, Pax6 is crucial for the differentiation of PECs, and its inactivation disturbs the adequate proportion of the different endocrine cell subtypes. Indeed, in *Pax6* KO mice [11, 12] and in *pax6b*^*−/−*^ zebrafish [13], the number of alpha, beta and delta pancreatic cells is significantly reduced while epsilon cells are increased. In the small intestine, Pax6 has been reported to be required for the differentiation of some EEC subtypes [14, 15]; however, it is still not known which set of genes is regulated by Pax6 during EEC and PEC differentiation and whether the Pax6-regulated network is shared by these two tissues.

EECs have been detected in the gut of many vertebrate and invertebrate species, and their formation appears early during animal evolution dating back to the cnidarian-bilaterian ancestor [16–18]. In contrast, the pancreas and the PECs appeared much later with the first vertebrate species [19, 20]. The similarity between EECs and PECs could be due to several reasons. The first possibility could be a common phylogenetic origin; the PECs could have indeed derived from some EECs and moved out from the gut to form clusters and to secrete other hormones like insulin and glucagon. This scenario is compatible with the “sister cell-type model” [21] where two cell types derived from an ancestral cell type by segregation of function (e.g. secretion of distinct hormones) due to the gain or loss of transcription factors. In this model, the two sister cell types still share many characteristics and can display similarities in their transcriptomes [22]. Another reason of the PEC and EEC similarities could be the co-option of the EEC (or neuronal) regulatory pathways by the pancreatic cells [23]. To approach this matter, we have measured in the present study the degree of similarity between EECs and PECs at the genome-wide expression level and determined whether they share regulatory programmes. We previously determined the transcriptomic landscape of zebrafish PECs [24]. In contrast, the EECs from zebrafish have not yet been fully characterized, and only a few hormones have been shown be expressed in these cells [25–29]. The first objective of this study was to perform a detailed characterization of zebrafish EECs by determining their transcriptomic profiles and identifying all neuropeptide hormones and regulatory genes expressed in these cells. Secondly, the transcriptomic landscape of zebrafish EECs was compared with those of zebrafish PECs to measure the degree of similarity. Finally, we analysed the consequences of *pax6b* inactivation on the transcriptome of both pancreatic and intestinal endocrine cells and show that Pax6b controls a large set of genes identical in both EECs and PECs further supporting their close relatedness. Finally, we discuss our findings in light of the evolution of EECs and pancreatic cells, supporting the model of a common phylogenetic origin and bringing some implications for deciphering the defects involved in type 2 diabetes.

## Results

### Characterization of the enteroendocrine cells in zebrafish

**Fig. 1.**
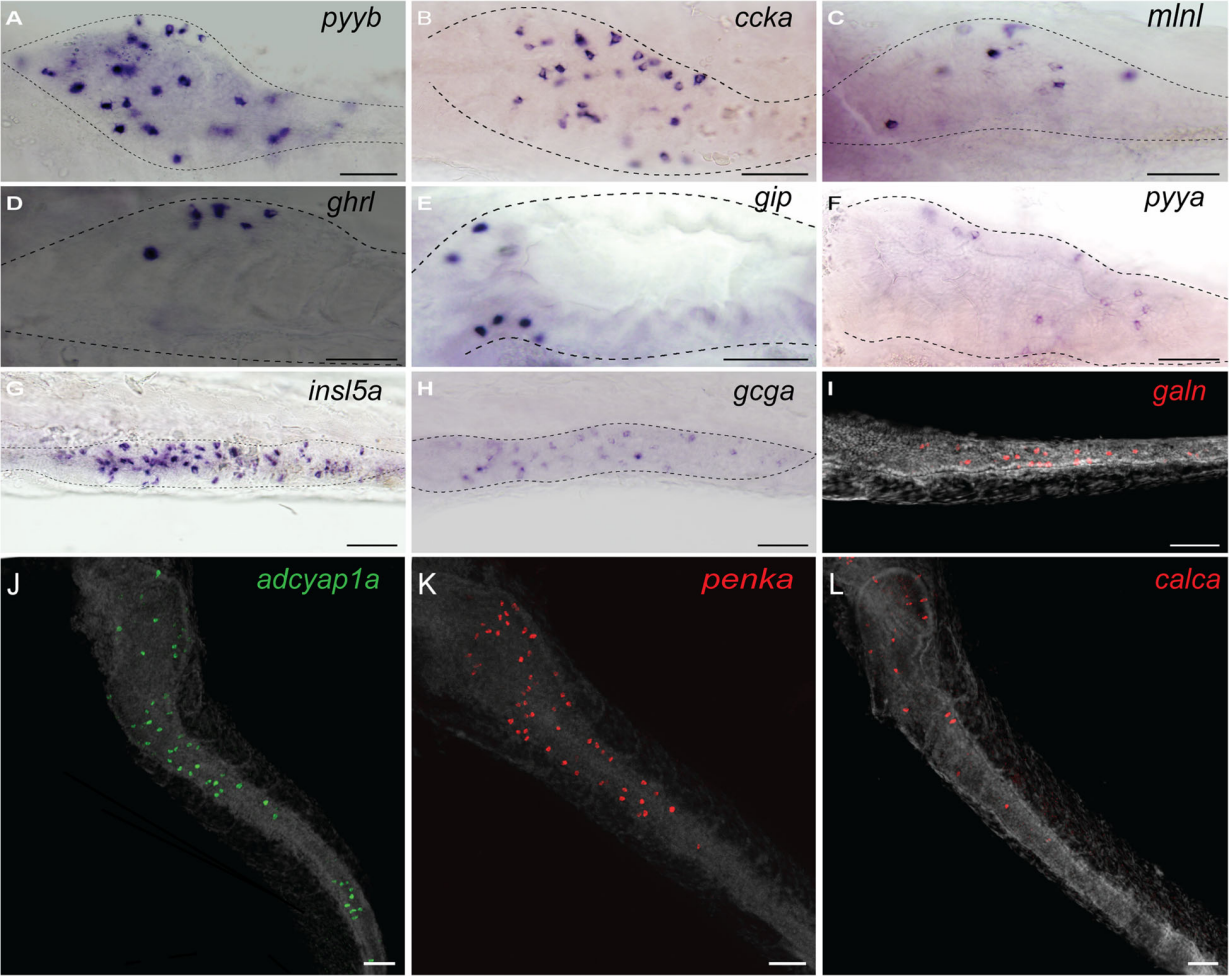
Expression of different enteroendocrine hormones in the zebrafish gut. Whole-mount in situ hybridization (WISH) obtained with different hormonal probes at 3 dpf (**a**–**h**, **k**) or 4 dpf (**i**, **k**). Ventral views of embryos with anterior on the left. The dotted lines represent the location of the gut. The probes used are as follows: *adcyap1a*, *adenylate cyclase-activating polypeptide 1a*; *ccka*, *cholecystokinin a*; *gcga*, *Glucagon a*; *pyya/b*, *Peptide YYa/b*; *ghrl*, *Ghrelin*; *gip*, *glucose-dependent insulinotropic polypeptide*; *insl5a*, *insulin-like peptide 5a*; *calca*, *calcitonin*. Scale bars, 50 μm. **i**–**l** Fluorescent in situ hybridization (FISH) (DAPI staining shown in grey)

In mice, at least 15 different hormones have been described to be expressed in the gut [1, 8]. Out of these, we identified in the zebrafish genome at least one orthologue for 11 of them, 9 showing syntenic conservation at their genomic loci with the murine/human genes, thereby validating orthology relationship (Table 1). We did not find any orthologous genes coding for gastrin (gast), secretin (sct), the pancreatic polypeptide (pp) nor motilin (mln); however, a *motilin-lik*e (*mlnl*) gene, proposed to be the functional equivalent of motilin, is present in the zebrafish genome [31]. In order to identify all peptide hormones expressed in the gut of zebrafish larva, we performed RNA-seq on isolated EECs. This was achieved by microdissection of the intestines from 4-dpf zebrafish transgenic *Tg(pax6b:GFP)*^*ulg515tg*^ larvae, which express GFP in the EECs [32], followed by cell dissociation and selection of GFP+ EECs by FACS. The restricted expression of Pax6b:GFP in the EEC and not in the enteric neurons (EN) was confirmed by immunohistochemistry showing a total absence of colocalisation between the Hu enteric marker and pax6b:GFP (Additional file 1: Fig. S1). RNA-seq was performed on four independent EEC preparations, and about 40 million paired-end reads were obtained from each Illumina library, 70–80% of which mapped to the zebrafish genome. We provide the expression level of all genes in counts per million (CPM) and in fragments per kilobase of exon model per million reads mapped (FPKM) in additional file 2: Table S1. This expression profiling allowed us to determine that, amongst the 12 zebrafish genes corresponding to EEC hormones, 11 are expressed in the zebrafish EECs at significant levels (above 100 CPM or 100 FPKM), *neurotensin* (*nts*) being the only hormone not expressed in EECs (Table 1). The expression of many of these hormones was further validated by whole-mount in situ hybridization (WISH) thereby locating the region of the gut displaying the strongest expression and defining the onset of their expression (Fig. 1 and Table 1). The number of EECs detected in the gut of 4-dpf zebrafish larvae by WISH varies for each hormone; for example, more than 20 cells expressing *peptide YY-b* (*pyyb*), *cholecystokinin-a* (*ccka*), *preproglucagon-a* (*gcga*), *galanin* (*galn*) or *insulin-like 5a* (*insl5a*) transcripts were detected (Fig. 1a, b, g–i; Table 1 and Additional file 3: Fig. S2) while only few cells express *ghrelin* (*ghrl*), *glucose-dependent insulinotropic polypeptide* (*gip*) and peptide YY-a (pyya) (Fig. 1d–f and Table 1). These differences of expression are also reflected in the RNA-seq data: *gcga*, *ppyb*, *ccka*, and *insl5a* reaching more than 2000 CPM while *ghrl* and *gip* range around 100 CPM (Table 1).

**Table 1:** Identification and expression level of zebrafish enteroendocrine hormones

	Mammalian hormones		Zebrafish hormones	Synteny	Onset time	Expression (WISH)			Cell number, mean ± SE	EEC RNA-seq (CPM)	EN RNA-seq (CPM)
						Bulb	Mid-gut	Posterior gut			
**A**	*1*	*Gcg*	*gcga*	Yes	3.5 dpf	**–**	✔	✔	33 ± 1.4	2058	57
			*gcgb*	Yes	N.T*					177	14
	*2*	*Sst*	*sst1.1*	Yes	**–**	**–**	**–**	**–**		20	13
			*sst1.2*	Yes	N.T*					88	1519
			*sst2*	Yes	3 dpf	✔	✔	**–**	2.8 ± 0.4	103	933
	*3*	*Ghrl*	*ghrl*	No	3 dpf	✔	**–**	**–**	2.1 ± 0.4	163	40
	*4*	*Pyy*	*pyya*	Yes	4 dpf	✔	**–**	**–**		437	1
			*pyyb*	Yes	3 dpf	✔	✔	**–**	41 ± 2.6	32,011	4
	*5*	*Cck*	*ccka*	Yes	3 dpf	✔	–	**–**	23 ± 3.0	6390	1
	*6*	*Gip*	*gip*	Yes	4 dpf	✔	**–**	**–**	2.4 ± 0.4	100	4
	*7*	*Vip*	*vip*	Yes	N.T*					0	511
			*vipb*	No	4 dpf	**–**	**–**	✔	N.T*	927	868
	*8*	*Nts*	*nts*	Yes	**–**	**–**	**–**	**–**		0	3
	*9*	*Insl5*	*insl5a*	No	4 dpf	**–**	✔	✔	39 ± 2.2	5602	14
			*insl5b*	No	N.T*					178	1
	*10*	*Nmb*	*nmba*	Yes	N.T*					45	116
			*nmbb*	No	3 dpf	✔	**–**	**–**	14 ± 2.0	2150	0
	*11*	*Galn*	*galn*	Yes	3.5 dpf	**–**	✔	✔	26 ± 1.8	824	157
	*12*	*Mln*	*/*	/	/	/	/	/		/	
			*mlnl*	No	3 dpf	✔	/	/	5.9 ± 0.7	708	18
	*13*	*Gast*	*/*	/	/	/	/	/		/	
	*14*	*Sct*	*/*	/	/	/	/	/		/	
	*15*	*Pp*	*/*	/	/	/	/	/		/	
**B**	*16*	*Adcyap1*	*adcyap1a*	No	3 dpf	✔	✔	✔	84 ± 6.9	6927	2
			*adcyap1b*	No	N.T*					6	251
	*17*	*Calca*	*calca*	Yes	4 dpf	✔	✔	**–**	12 ± 0.7	2161	1
	*18*	*Penk*	*penka*	Yes	3 dpf	✔	✔	**–**	68 ± 3.6	12,544	2
			*penkb*	Yes	N.T*					2	

(Upper A panel, lanes 1–15) Identification of the zebrafish orthologues for the 15 mammalian genes coding for enteroendocrine hormones by “in silico” search of the zebrafish genome. (Lower B panel, lanes 16–18) Identification of the 3 novel hormones expressed in zebrafish EECs by screening the RNA-seq data with the Gene Ontology term “hormone activity” (GO: 0005179) and selecting hormones expressed at high levels (> 2.000 CPM). Column 3 indicates the Ensembl ID of the zebrafish genes. Column 4 displays whether a synteny conservation is observed between mouse and zebrafish loci. Column 5 indicates the expression onset time determined by WISH for each hormone transcripts and their localization in the zebrafish gastrointestinal tract (column 6). Bulb corresponds to the enlarged rostral part of the intestine; the mid-gut is located posterior to the bulb and contains the majority of goblet cells at 5 dpf; the posterior gut corresponds the most caudal part of intestine containing few goblet cells [29]. Column 7 indicates the mean (± standard errors (SE)) of the EEC numbers detected by WISH at 4 dpf. The number of EEC counted for each individual larvae is provided in Fig. S2. N.T*: not tested; the number of *vipb*+ EEC cells could not be quantified as most of the *vipb*+ cells detected in the gut are *vipb*+ enteric neurons. Column 8 indicates the expression levels determined by RNA-seq of EEC in CPM (counts per million). Column 9 indicates the expression levels determined by RNA-seq of enteric neurons in CPM [30]. –: not detected by WISH; ✔: detected by WISH

*Gcg* glucagon, *Sst* somatostatin, *Ghrl* ghrelin, *Pyy* peptide YY, *Gast* gastrin, *Ckk* cholecystokinin, *Gip* glucose-dependent insulinotropic polypeptide, *Vip* vasoactive intestinal peptide, *Mln* motilin, *mlnl* motilin-like, *Pp* pancreatic polypeptide, *Nts* neurotensin, *Sct* secretin, *Insl5* insulin-like peptide 5, *Nmb* neuromedin B, *Adcyap1* adenylate cyclase-activating polypeptide 1, *Galn* galanin, *Calca* calcitonin

**Fig. 2.**
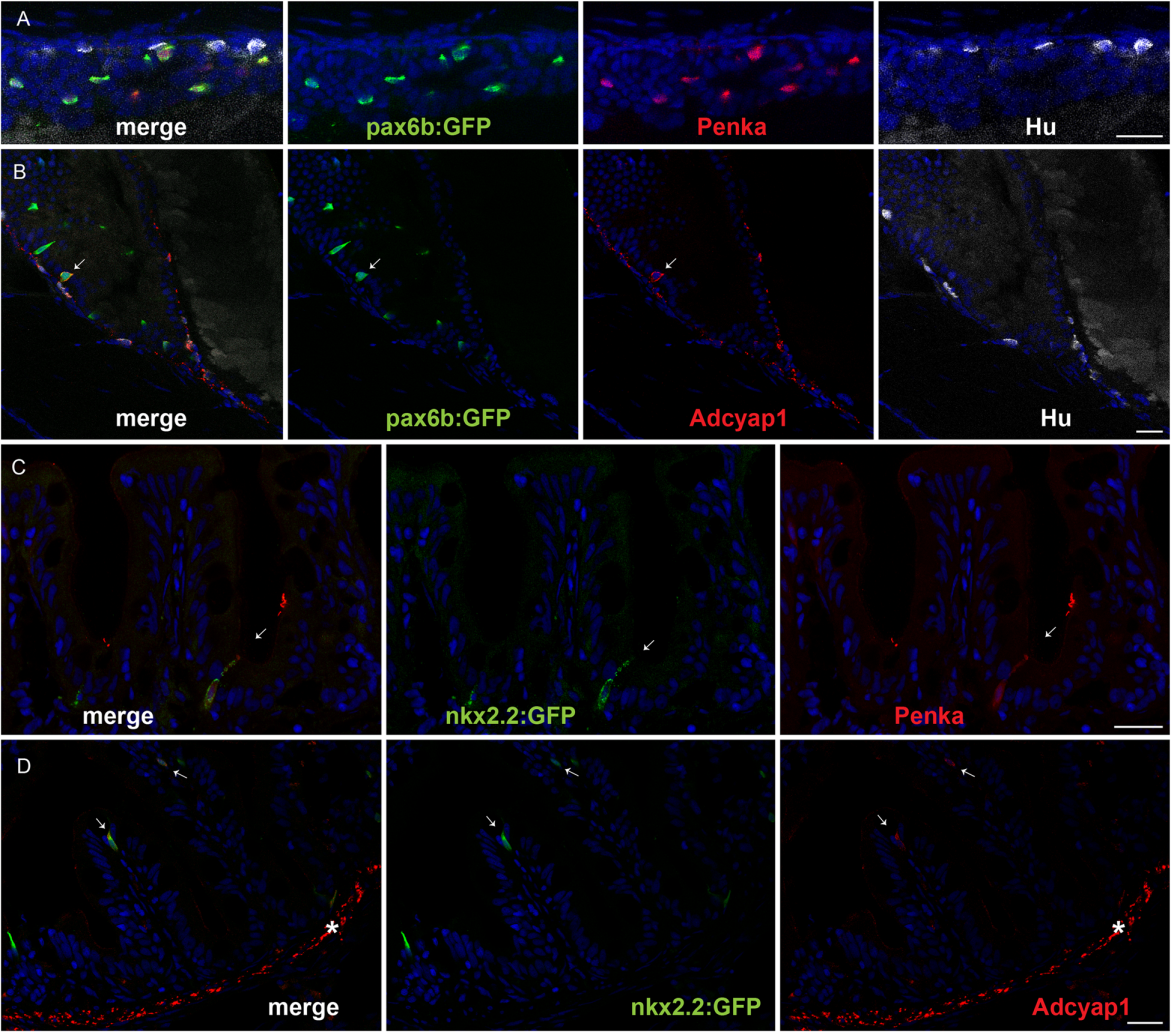
Immunostaining of enkephalin and Adcyap1 in the zebrafish intestine. **a**, **b** Confocal images of the intestines from 5-dpf *Tg(pax6b:GFP)* zebrafish larva stained with antibodies directed against GFP (green), Hu (white), ENKEPHALIN (**a**; red) or ADCYAP1 (**b**; red). **c**, **d** Confocal images of the intestine of adult *Tg(8.5nkx2.2:GFP)* stained with antibodies directed against GFP (green), ENKEPHALIN (**c**; red) or ADCYAP1 (**d**; red). Arrows show the location of some EECs expressing Adcyap1 (**b**, **d**) or enkephalin (**c**). Adcyap1 immunostaining is also observed at the level of EN axon fibres in gut larva (**b**) and in the muscularis layer of the adult intestine (shown by an asterisk in **d**). The zebrafish Adcyap1a and Adcyap1b display respectively 6 and 3 mismatches with human ADCYAP1 at the level of the epitope reacting with the Adcyap1 antibody, explaining the stronger signal of Adcyap1b in EN fibres. (The nkx2.2:GFP transgene is expressed specifically in EECs like Pax6b). Scale bar, 20 μm

Data mining of the zebrafish EEC RNA-seq with the GO term “hormone” also revealed a strong expression of three neuropeptide transcripts not described so far as expressed in mammalian EECs: *adenylate cyclase-activating polypeptide 1a* (*adcyap1a*) (previously named *PACAP*), *proenkephalin-a* (*penka*) and *calcitonin* (*calca*) (Table 1). Fluorescent in situ hybridization experiments (FISH) confirmed the expression of these transcripts in the zebrafish EECs (Fig. 1j–l). An average of 68 *penka+* and 12 *calcitonin+* EECs were detected in the bulb and the mid-gut and more than 80 cells expressing *adcyap1a* were found all over the gut (Table 1 and Additional file 3: Fig. S2). As enteric neurons (ENs) surround the gut at that stage and express a variety of neuropeptides, some being expressed also by EEC, we next determined whether some of the hormone expressed by EEC would be also detected in ENs. For that purpose, we retrieve the transcriptomic data of zebrafish ENs, recently determined by RNA-seq after sorting the ENs by FACS from *Tg(phox2b::GFP)* larvae at 7 dpf [30]. Comparison of the EN and EEC RNA-seq data indicates that most EEC hormones display a much higher expression in EECs compared to ENs, except for *sst1.2*, *sst2* and *vipb* (see Table 1). FISH using the EN-specific marker *Phox2b* confirms the strong expression all over the gut of *vipb* in ENs while the *vipb*+ EECs are mostly located in the posterior gut (Additional file 4: Fig. S3A and B). Interestingly, RNA-seq data indicate that adcyap1a is exclusively expressed by EECs while adcyap1b is restricted to ENs. FISH confirms the strong expression of adcyap1a in EECs and not in *phox2b*+ ENs (Additional file 4: Fig. S3 C). However, IHC using an anti-ADCYAP1 antibody reveals staining in pax6b:GFP+ EECs as well as at the level of EN axon fibres in larvae, as previously shown [33] (Fig. 2b). Similar results were observed in the adult intestine (Fig. 2d). Taken together, all these data confirm the expression of the Adcyap1a in EECs and of Adcyap1b in ENs. As for the two other new hormones Calca and Penka, RNA-seq comparison indicates that they are exclusively expressed by EECs, and this was confirmed for Penka for which we observe strong enkephalin immunostainings in EECs but not in ENs at the larva stage as well as in adults (Fig. 2a, c).

### Close relatedness between the enteroendocrine and pancreatic endocrine cells

**Fig. 3.**
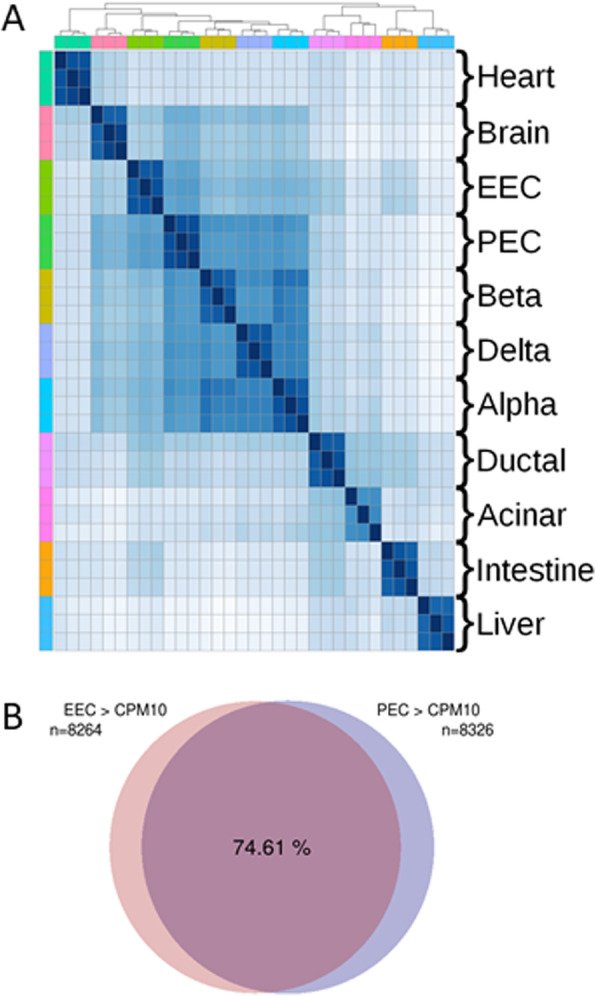
Similar gene expression profiles between zebrafish EECs and PECs. Global comparison of transcriptomic profiles (RNA-seq) from distinct zebrafish tissues/organs. **a** Clustered heatmap displaying the Euclidean distance matrix between every pair of zebrafish RNA-seq datasets. The order of the different tissues is identical for the two axes (rows and columns) as shown by the different colours on the upper part and left part of the matrix. Each zebrafish tissue (analysed in triplicate) was compared with the other samples as presented in the matrix. Darker colour indicates closer distance (i.e. more similar transcriptomes). EEC transcriptome is mostly similar to the transcriptome of embryonic pancreatic endocrine cells (PECs) or to the adult alpha, beta or delta pancreatic cells. The accession number of all used RNA-seq datasets is given in the “Methods” section. **b** Venn diagram showing the overlap (74%) of genes expressed in both EECs and PECs above the threshold of 10 CPM. A total of 8264 genes and 8326 genes were expressed above 10 CPM (counts per million) respectively in EECs and in PECs

Although EECs and PECs are known to share common features, we wanted to evaluate the degree of similarity at a more global level. Thus, we compared the RNA-seq data of the zebrafish PECs and EECs as well as of other zebrafish cell types and organs used as control (Fig. 3a). The heatmap shows that all endocrine cells cluster together apart from other pancreatic or intestinal cells and other tissues. Indeed, the EEC cluster is much closer to the PEC cluster than to the ductal or the acinar pancreatic cells or even to the whole intestine tissue which is mainly composed of enterocytes and of only 1–2% of EECs. In agreement with previous reports showing shared features between pancreatic cells and neurons [23, 34], this clustering analysis reveals also transcriptomic similarities of brain tissue not only with pancreatic but also with intestinal endocrine cells.

**Fig. 4.**
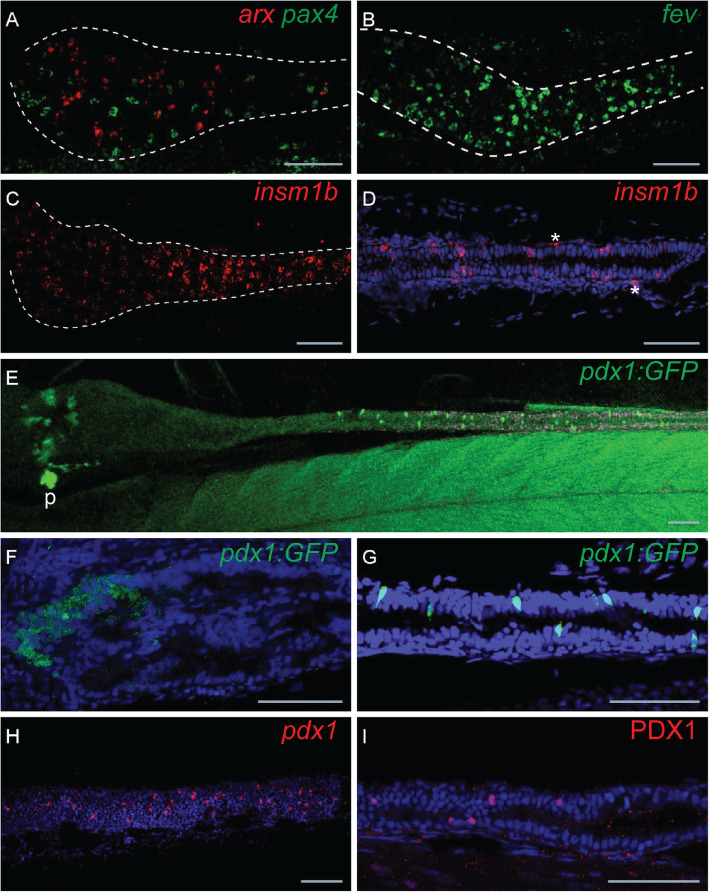
Expression profiles of *pax4*, *arx*, *pdx1* and *fev* in the zebrafish gut. Fluorescent in situ hybridization (FISH) performed on 66 hpf (**a**), 72 hpf (**b**–**d**) or 96 hpf (**e**–**i**) embryos. Views of the gut (delimited by dotted lines; anterior to the left) showing the non-overlapping expression of *pax4* (green) and *arxa* (red) (**a**), numerous EECs expressing *fev* (**b**) and *insm1b* (**d**). Higher magnification showing *insm1+* EECs located within the gut epithelium and *insm1+* enteric neurons (shown by asterisks) outside but juxtaposed to the epithelium. **e** View of the gut from the tg(BAC pdx1:GFP) larvae (GFP immunostaining) with higher magnification of the anterior (**f**) and posterior (**g**) parts. FISH using *pdx1* probe (**h**) and immunostaining using Pdx1 antibody. P, pancreas. Scale bars, 20 μM

The close relatedness of EECs and PECs is also illustrated by the high percentage of genes expressed in both tissues: amongst all genes expressed in pancreatic or intestinal endocrine cells above the threshold of 10 normalized CPM (8326 and 8264 genes, respectively), 74% of them are expressed in both tissues (Fig. 3b), while if the same analysis is done for genes expressed in EECs and liver, only 45% of them are expressed in both tissues (data not shown). Similar percentages are obtained when considering genes coding for transcription factors (TF): 72% of all expressed TF-coding genes are detected in both PECs and EECs, while only 45% are expressed in both pancreatic endocrine cells and liver. The list of 481 TF expressed in both PECs and EECs (given in Additional file 5: Table S2) includes notably most of the transcription factors reported to be important for zebrafish PEC differentiation (Table 2). For example, *arxa* and *pax4*, known to be an important determinant of cell fate of pancreatic endocrine cells [35, 36], are also expressed in the zebrafish gut, and like in pancreas, their expression is mainly non-overlapping (Fig. 4a). *Fev*, known to be highly expressed in pancreatic endocrine cells [37], is also amongst of the most highly expressed transcription factors in the zebrafish EECs (Table 2) and is detected by FISH in many cells all over the gut (Fig. 4b). Similarly, *insm1b* [9] is also highly expressed in zebrafish EECs (Fig. 4c, d and Table 2) in addition to its expression in enteric neurons (labelled with asterisk in Fig. 4d) as described in mice. Expression of *pdx1*, visualized using the *tg(BAC pdx1:GFP)* [38], is also detected in scattered EECs of the mid- and posterior gut (Fig. 4e, g), in addition to a widespread expression in cells of the rostral part of the intestine as previously described [39] (Fig. 4e, f). The expression of *pdx1* in EECs of the mid- and posterior gut was confirmed by WISH (Fig. 4h) and by immunohistochemistry (Fig. 4i). As expected, *ascl1a* was barely detected in the transcriptome of 4-dpf EECs (Table 2) as we have previously shown that its expression in the secretory precursor cells turns off as soon as the cells pursue their differentiation process [27]. In the same way, *sox4b*, coexpressed with *ascl1a* in the secretory precursor cells, is hardly detected in the transcriptome of the EECs at 4 dpf (Table 2), indicating that *sox4b* is also switched off with the maturation of the EECs. In contrast, Nkx6.1, Nkx6.2 and Mnx1 are the only 3 transcription factors involved in zebrafish PEC differentiation that are not expressed in EECs. Many of these EEC/PEC transcription factors are expressed at lower levels in enteric neurons as revealed by the EN RNA-seq data (Table 2) with the exception of insm1b, neurod1 and ascl1a which are expressed above 50 CPM.

**Table 2:** Expression level in EECs of transcription factors involved in zebrafish PEC differentiation

Pancreatic endocrine TF	Onset time in the gut	Expression in the gut			EEC RNA-seq (CPM)	EN RNA-seq (CPM)
		Bulb	Mid-gut	Posterior gut		
*ascl1b*	**–**	**–**	**–**	**–**	2	3
ascl1a*	36 hpf	✔	✔	✔	25	55
sox4b*	38 hpf	✔	✔	✔	36	22
*insm1b*	≤ 52 hpf**	✔	✔	✔	638	189
*insm1a*	≤ 52 hpf**	N.D	N.D	N.D	123	38
*foxo1a*	≤ 52 hpf**	✔	✔	✔	504	33
neurod1*	52 hpf	✔	✔	✔	474	67
nkx2.2a*	52 hpf	✔	✔	✔	195	2
*pax4*	58 hpf	✔	✔	✔	225	0
pax6b*	60 hpf	✔	✔	✔	826	23
rfx6*	62 hpf	✔	**–**	**–**	13	8
*fev*	62 hpf	✔	✔	✔	951	3
isl1*	65 hpf	✔	✔	✔	134	31
*arxa*	66 hpf	✔	✔	✔	54	1
*pdx1*	72 hpf in EEC	**–**	✔	✔	614	4
*nkx6.1*	**–**	**–**	**–**	**–**	1	2
*nkx6.2*	**–**	**–**	**–**	**–**	5	1
*mnx1*	**–**	**–**	**–**	**–**	1	0

Recapitulation of the WISH data performed on zebrafish embryos from 36 to 72 hpf showing the expression onset time and the distribution profile in the gut determined by WISH for 17 transcription factors known to be involved in pancreatic endocrine cell (PEC) differentiation. **Based on RNA-seq data from FACS-sorted sox17:dsRED cells of the microdissected intestine at 52 hpf, *insm1a*, *insm1b* and *foxo1a* are already expressed in the gut at 52 hpf (Reuter et al., article in preparation). The two last columns indicate the expression level in EECs and in enteric neurons (ENs) as determined by RNA-seq in normalized CPM. (N.D.: not determined; −: not detected by WISH; ✔: detected by WISH). All these TF are expressed at significant levels in EEC, except *ascl1b*, *nkx6.1*, *nkx6.2* and *mnx1*. Expression data of genes labelled with an asterisk are from [27]

The number of genes which are selectively expressed in PECs or in EECs is relatively low (gene lists available in Additional file 6: Table S3). Gene Ontology (GO) enrichment analyses of the PEC-specific genes highlight terms such as “cell adhesion” due to the enriched expression of many cadherins and other cell adhesion molecules. This enrichment makes senses as the PECs are clustered into islets while EECs are scattered in the gut. The EEC-specific genes display an enrichment of GO terms including “drug transmembrane transport”, “ABC transporter” or “oxidation process” due to the enriched expression of many transmembrane transporters and cytochrome P450 type molecules (Additional file 6: Table S3).

In conclusion, all these data indicate a close relatedness between PECs and EECs at the level of their transcriptomes and of their regulatory factors, suggesting the involvement of similar regulatory programmes in these two cell types.

#### Shared Pax6-dependent regulatory programmes between pancreatic and intestinal endocrine cells

**Fig. 5.**
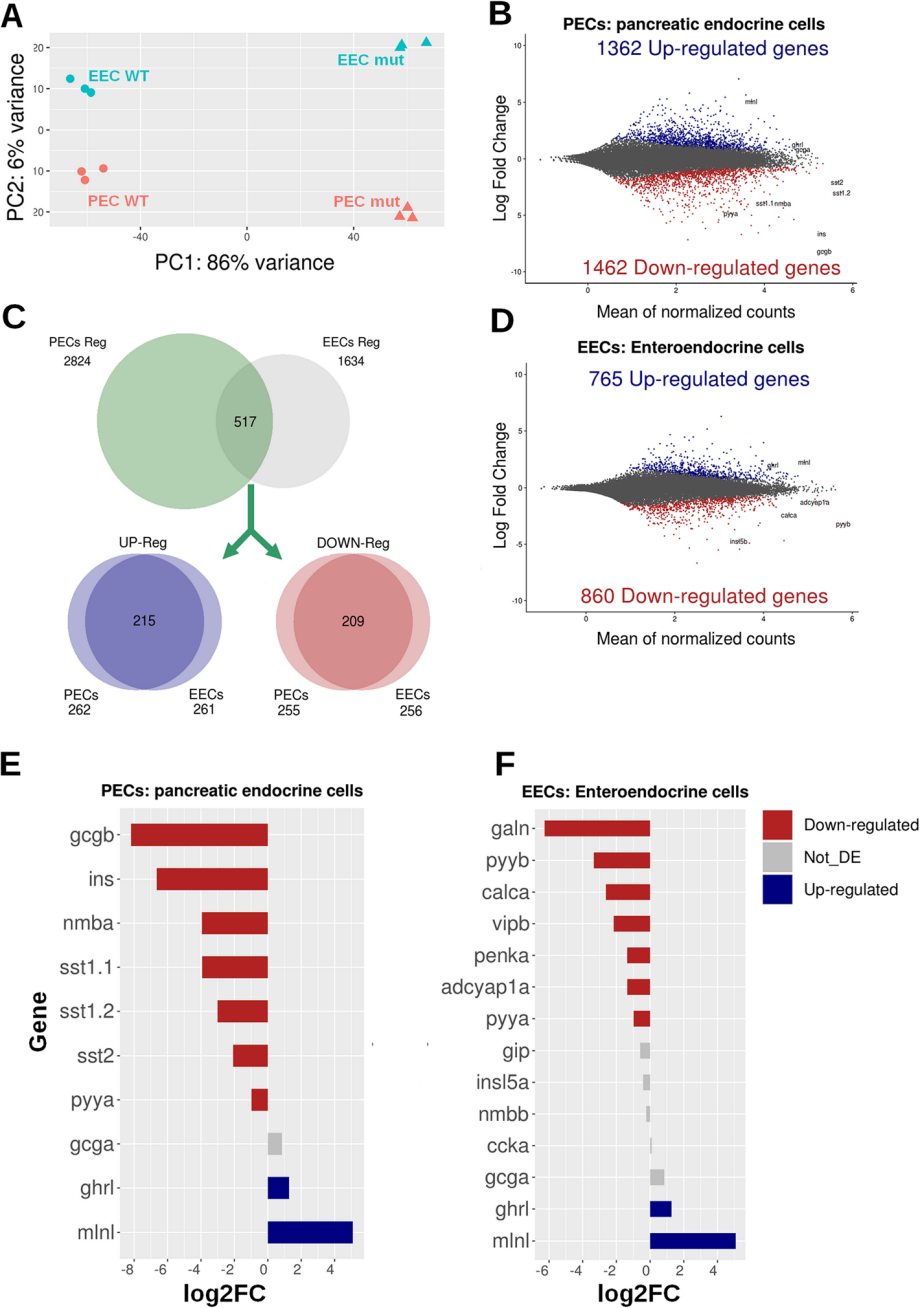
RNA-seq analysis of EECs and PECs from wild-type and *pax6b* mutants identifies a set of genes displaying similar Pax6b regulation in both the pancreas and intestine. **a**, Principal component analysis (PCA) on all EEC and PEC RNA-seq data obtained from wild-type and pax6b^sa0086^ mutants. The close clustering of the triplicate wild-type and mutant samples demonstrates the high reproducibility of the data. **b**, **d** MA plots showing all the upregulated (in blue) and downregulated (in red) genes in the *pax6b*^*sa0086*^ mutant PECs (**b**) and EECs (**d**). **c** Venn diagrams showing the overlap of genes regulated in both EECs and PECs; amongst the 517 regulated genes, 215 and 209 genes are respectively up- and downregulated in both cell types as shown in the blue and red Venn diagrams. **e**, **f** Expression ratio (in log2 of fold change from *pax6b* mutant versus wild-type) of hormones expressed in PECs (**e**) and in EECs (**f**); downregulated hormones are in red, upregulated hormones are in blue and not statistically are affected in grey; RNA-seq values are shown in Tables 3 and S4

To investigate whether pancreatic and intestinal endocrine cells are controlled by similar regulatory programmes, we analysed the effect of Pax6 inactivation on the transcriptome of both cell types. Zebrafish has two *pax6* paralogs; however, only *pax6b* is expressed in PECs and EECs. To isolate *pax6b* mutant EECs and PECs, *pax6b*^*sa0086*^ heterozygous fish harbouring the *Tg(pax6b:GFP)* transgene were inbred and *pax6b*^*sa0086*^ homozygous embryos were selected based on lens abnormalities [13]. The expression of the transgene *Tg(pax6b:GFP)* was not perturbed in the homozygous mutants (data not shown), allowing us to isolate by FACS the PECs or EECs from *pax6b*^*−/−*^ mutant larvae after microdissection of the dorsal pancreatic bud or intestine, respectively. RNA-seq was performed on three independent preparations of EECs or PECs purified from *pax6b*^*sa0086*^ mutant and from wild-type zebrafish (see the “Methods” section). The principal component analysis shows a tight clustering of replicates underscoring a good reproducibility of the data and showing distinct clusters for mutant and wild-type cells for both PECs and EECs (Fig. 5a). Differential expression analyses identified 2824 and 1634 Pax6b-regulated genes in PECs and in EECs, respectively (FDR < 0.1) (Fig. 5b, d) (expression of all genes and of Pax6b-regulated genes are given respectively in Additional files 7 and 8: Table S4 and S5). Comparison of these two sets of genes reveals that there is a large set of 517 genes which are altered by *pax6b* inactivation in both organs (Fig. 5c); this overlap is statistically significant according to Fisher’s exact test (*p* value < 2.2e−16). Furthermore, most of these genes (424 genes) are regulated in the same way in the pancreas and in the intestine (215 upregulated and 209 downregulated genes) (Fig. 5c), indicating that a part of the Pax6-dependent gene regulatory network is thus identical in EECs and PECs. To explore the pathways and biological processes regulated by Pax6b, we performed a Gene Ontology analysis on these 3 sets of Pax6b-regulated genes: the common set for PECs and EECs (517 genes) or the specific one for PEC (2307 genes) or EECs (1117 genes) (see Additional file 8: Table S5). GO terms associated to “cell-cell signalling”, “regulation of appetite” or “hormone activity” were enriched significantly in the common set as several hormones expressed in PECs and EECs are affected in the pax6 mutant (e.g. *pyya*, *sst2*, *pyyb*, see below). Such an effect results probably from a change in the expression of several transcription factors (e.g. *pdx1*, *neurod1*, *rfx6*, *nkx2.2*, see below) as revealed in the GO lists “endocrine pancreas development” and “sequence-specific DNA binding”. Also, the GO analysis highlights the role of Pax6b in the control of ion transport (K+, Ca++ and Cl−) as well as calcium signalling. Interestingly, “ion transport” and “calcium channel/binding activity” were also GO-enriched terms amongst the genes regulated by Pax6b only in PECs or in EECs, supporting an important role of Pax6b for controlling these pathways.

#### Drastic increase of ghrelin/motilin-like-expressing cells in pax6b^−/−^ pancreatic and intestinal endocrine cells with a concomitant decrease of other endocrine cell subtypes

**Fig. 6.**
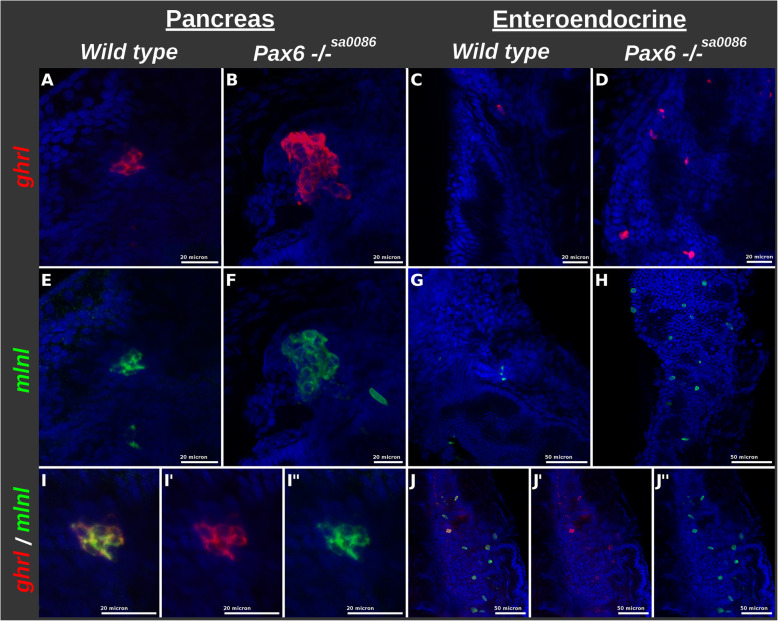
Increase in the number of cells co-expressing *ghrl* and *mlnl* in the *pax6b*^*−/−*^ PECs and EECs. FISH using ghrelin (ghrl, labelled in red) and motilin-like (mlnl, labelled in green) probes on 2.5 dpf (**A**, **B**, **E**, **F** and **I**) and 4.5 dpf (**C**, **D**, **G**, **H**, **J**) zebrafish larvae. Confocal views of the pancreatic islet (**A**, **B**, **E**, **F**) and of the gut (**C**, **D**, **G**, **H**). **I**, **J** Overlays of *ghr*l (**I′** and **J′**) and *mlnl* (**I″** and **J″**) stainings demonstrating co-labelling with the two probes. **A**, **E**, **I**, **I′**, and **I″** are views of the pancreatic islet from a wild-type larva while **B** and **F** are the pancreatic islet from *pax6b*^*−/−*^. **C**, **G** Views of the intestines from wild-type larvae; **D**, **H**, **J**, **J′**, **J″** Views of the gut from a *pax6b*^*−/−*^ larvae. Scale bars: 20 microns for **A-F** and **I** panels, 50 microns for **G**, **H** and **J** panels

**Fig. 7.**
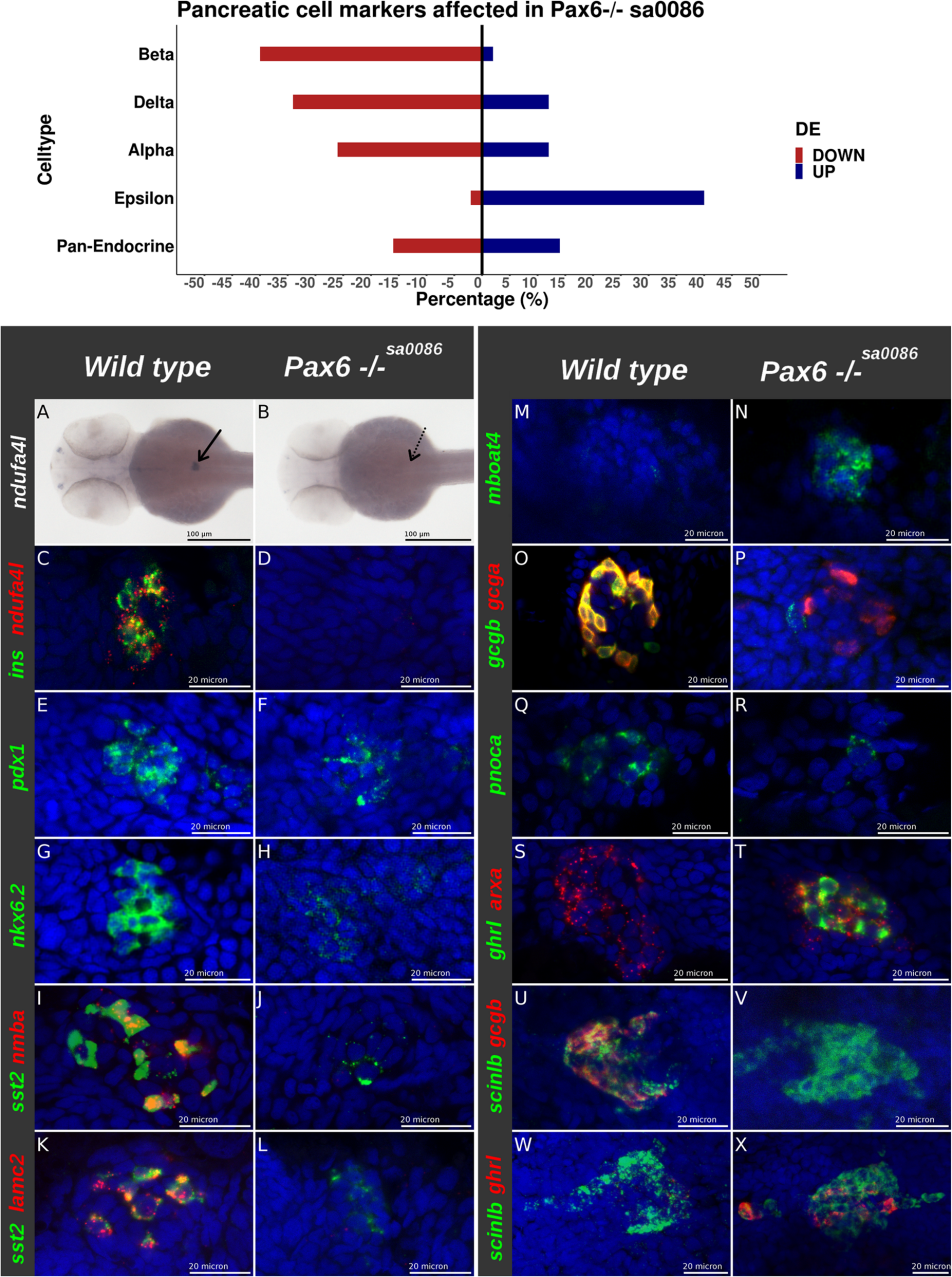
*pax6b* inactivation leads to a loss of pancreatic beta cells, reduction of delta cells, increase of epsilon cells and abnormal alpha cells. (Upper panel) percentage of markers from beta-, delta-, alpha-, epsilon- and pan-endocrine cells which are downregulated (in red) or upregulated (in blue) in the pax6b^−/−^ PECs. The list of markers for each cell subtype is shown in Additional file 9: Table S6. The up- or downregulation was determined from RNA-seq data from pax6b^sa0086^ mutant versus wild-type. (Lower panel) Expression analysis of cell subtype markers by WISH (**a**, **b**) and FISH (**c**–**x**) on 2 dpf zebrafish *pax6b* mutant and wild-type embryos as noted on the top of the panel; probes are indicated on the left of each picture. Scale bars: 100 µm for panels **a** and **b** and 20µm for panels **c** to **x**

The above analyses indicate a regulation by Pax6 of the expression of several hormones in PECs and EECs. Amongst them, *ghrelin* (*ghrl*) and *motilin-like* (*mlnl*) genes are strongly upregulated in both the pancreas and intestine of *pax6b* mutants (Fig. 5e, f and Table 3). FISH experiments confirmed an increase in the number of *ghrl+* cells and *mlnl+* cells in both the pancreas and intestine (Fig. 6A–H). Furthermore, double fluorescent staining demonstrated that these two hormones are often co-expressed in the same endocrine cells (Fig. 6I, J). In both tissues, the increase of *ghrl* and *mlnl* gene expression is concomitant to a decrease of other hormones: *galn*, *pyyb* and *calca* genes are indeed significantly downregulated in EECs of *pax6b*^*sa0086*^ mutants, while in the pancreas, the expression of notably the *insulin* (*ins*) and *somatostatin-1.1*, *1.2* and *2* genes is strongly decreased (Fig. 5e and Table 3). To determine whether these modifications in hormone expression result from a change in cell subtype proportion in *pax6b* mutant PECs, we analysed the expression of markers of these different pancreatic cell subtypes that we and others have previously identified (Additional file 9: Table S6) [24, 40]. We found that about 40% of genes showing an enriched expression in beta cell (with a fold enrichment > 4-fold) were downregulated in the islet of *pax6b*^*−/−*^ embryos, while only 2% were upregulated (Fig. 7, upper panel), strongly suggesting a loss of beta cells. This loss was further confirmed by FISH experiments: firstly, *ndufa4l2*, a gene showing a restricted expression in zebrafish pancreatic beta cells, was not detected anymore in the *pax6b* mutants (Fig. 7a–d). Similarly, the expression of the two beta cell-enriched transcription factors *pdx1* and *nkx6.2* were strongly decreased in PECs of *pax6b* mutants while could still be detected at low levels in the ventral pancreatic progenitors (Fig. 7e–h, Table 3). All these data indicate the loss of beta cells in *pax6b*^−/−*sa0086*^ embryos*.* Concerning the pancreatic delta cells, 35% of delta-enriched genes were downregulated in the *pax6b*^*sa0086*^ mutants and 12% were upregulated (Fig. 7, upper panel). The decreased number of delta cells in *pax6b* mutants was further supported by FISH through the strong reduction of cells expressing *somatostatin2* (*sst2*) as well as the loss of the delta-specific markers *laminin C2* (*lamc2*) and the hormone peptide *Neuromedin Ba* (*nmba*) (Fig. 7i–l, Table 3). The two delta-specific transcription factors Cdx4 and Hhex were also downregulated in PECs of *pax6b* mutants (Table 3). Inversely, 40% of genes with enriched expression in pancreatic epsilon cells are upregulated in *pax6b* mutants while only 2% are decreased (Fig. 7, upper panel). Like *ghrelin* and *motilin-like*, *mboat4*, another epsilon-specific marker, is strongly stimulated following *pax6b* inactivation as revealed by FISH (Fig. 7m, n) and by the RNA-seq data (Table 3). Thus, these data also indicate an increase of epsilon cells in *pax6b*^*sa0086*^ mutant embryos. Finally, the alpha cells in pax6b mutants display an “ambiguous” phenotype with a respective decrease and increase of 26% and 12% of alpha enriched genes (Fig. 7, upper panel). The RNA-seq data indeed reveal a strong decrease of the alpha cell markers *gcgb* and *pnoca* while others like *gcga*, *arx* and *scinlb* are even increased (Table 3). FISH confirmed these results. Indeed, although *gcga* and *gcgb* are detected in the same alpha cells in wild-type embryos, *gcgb* expression was severely reduced in the *pax6b* mutants in contrast to *gcga* that remained highly expressed (Fig. 7i, j). Like *gcgb*, the alpha cell marker *pnoca* (*prepronociceptin a*) was drastically downregulated (Fig. 7q, r). On the other hand, the alpha cell marker *scindl* (Fig. 7u, v) and the alpha cell determinant *arxa* (Fig. 7s, t) were still expressed. Double labelling of *arxa* and *scinlb* with *ghrelin* revealed that these two markers are actually expressed in both alpha and epsilon cells in zebrafish (Fig. 7s–x). Regarding the pan-endocrine markers, which are not enriched in a specific endocrine cell subtype, only a minority (about 15%) are either down- or upregulated in the pax6b^−/−^ mutants (Fig. 7, upper panel). Altogether, our data indicate that there is a loss of beta cells and a strong reduction of delta cells in the *pax6b*^−/−^ mutants with a concomitant increase in the number of epsilon cells. Alpha cells are still generated in the *pax6b*^*sa0086*^ mutants, but these cells are misdifferentiated.

**Table 3:** Expression level of some selected markers in EEC and PEC isolated from wild-type and pax6b^sa0086^ mutant larvae

Markers	Gene	PEC wild type	PEC pax6b^**−/−**^	EEC wild type	EEC pax6b^**−/−**^
Epsilon PEC	***ghrl***	996	3156	184	876
	***mlnl***	7.6	385	785	4604
	***mboat4***	0.7	201	0.02	1.3
Beta PEC	***ins***	13,111	112	1.1	2.9
	***ndufa4l2a***	1729	82	1.8	1.2
	***nkx6.2***	330	10	5.5	7.7
	***pdx1***	1377	37	453	46
Delta PEC	***sst2***	24,070	6287	107	440
	***sst1.1***	668	37	27	0.2
	***sst1.2***	33,282	4375	89	37
	***lamc2***	153	16	4	31
	***nmba***	1640	110	46	15
	***hhex***	95	27	0.5	0.1
	***cdx4***	181	64	40	32
Alpha PEC	***gcgb***	15,002	42	179	2.4
	***gcga***	1776	3791	1982	1526
	***pnoca***	144	14	11	2.2
Alpha and epsilon	***scinlb***	83	1374	476	1365
Alpha and epsilon	***arxa***	67	290	42	57
EEC hormones	***galn***	0.05	0.19	758	12
	***pyyb***	32	20	34,323	3746
	***calca***	57	24	1933	349
	***adcyap1a***	0.58	0.41	6201	2672
	***vipb***	0.07	0.17	737	190
	***ccka***	0.43	1.01	6257	7212

The expression level (given in normalized CPM) was obtained from the RNA-seq data. The values are the expression mean of triplicate samples. Table S4 provides the expression level of all genes (values for each sample, means and standard deviation)

**Fig. 8.**
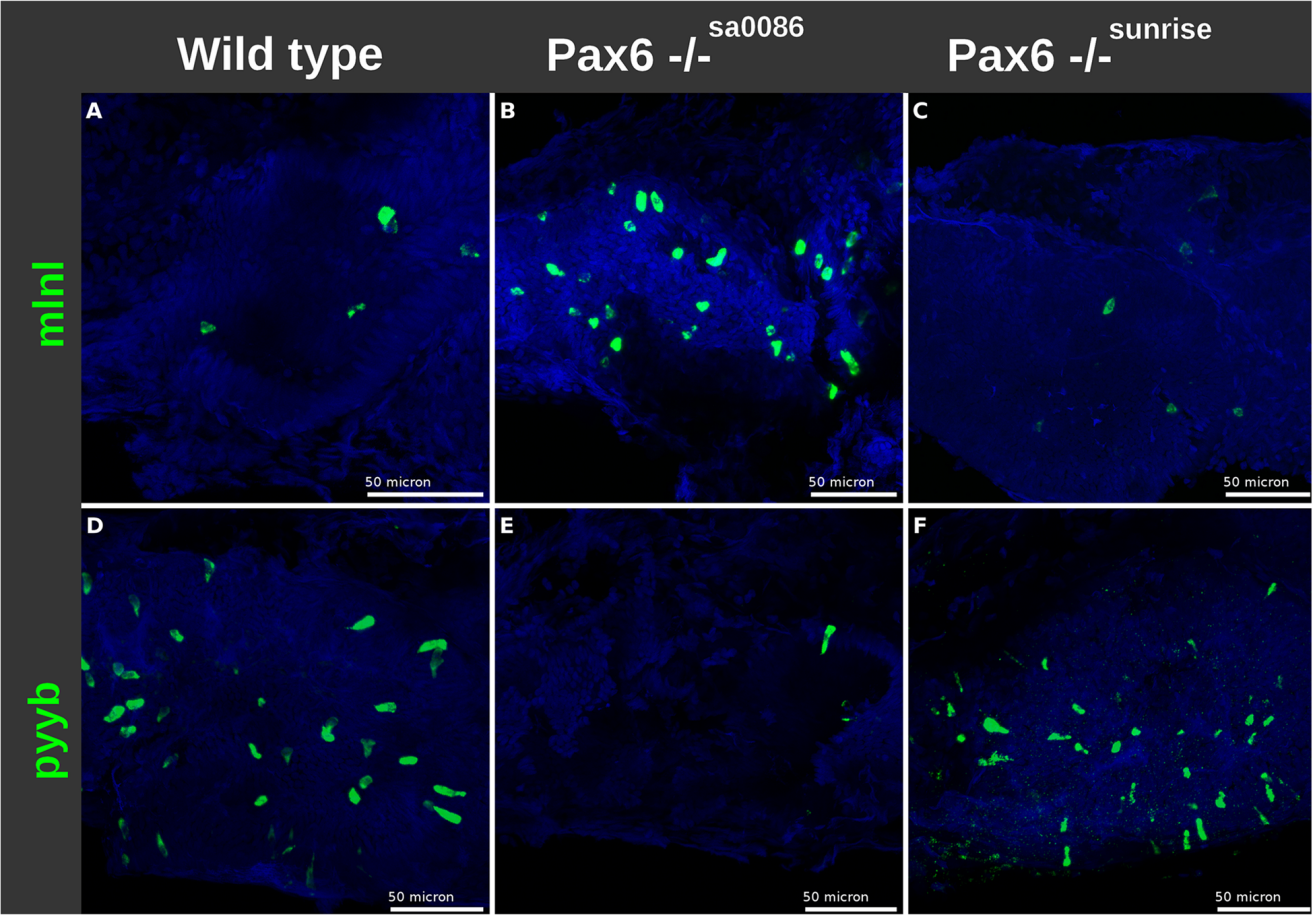
The effect of Pax6b on the number of EEC expressing *pyyb* and *mlnl* does not depend on its homeodomain. FISH showing the respective increase of *mlnl*+ EECs and decrease of *pyyb*+ EECs in the *pax6b*^*sa0086*^ null mutants (**b**, **e**), while the hypomorphic *pax6b*^*sunrise*^ mutants harbouring a mutation in *pax6b* homeobox do not display modifications in the expression of EEC hormones (**c**, **f**)

To verify that the increase of *ghrl+/mlnl+* endocrine cells in the intestine of *pax6b*^−/−^ larvae (Fig. 6) is also concomitant to a decrease of other EECs, we analysed by FISH the expression of *pyyb*, a gene highly expressed in the intestine and strongly downregulated in *pax6b* mutants (Table 3). The number of EECs expressing *pyyb* was indeed drastically reduced in the intestine of *pax6b*^*sa0086*^ mutants (Fig. 8d, e). Thus, all these FISH confirms that *pax6b* has a similar role in the control of the proportion of the various endocrine cell types in both intestine and pancreas.

#### Like in the pancreas, the homeodomain of Pax6b is not crucial for the differentiation of the endocrine cells of the intestine

Previous studies have shown that a missense mutation in the homeobox of *pax6b* (sunrise allele) gene causes eye developmental defects but does not perturb the development of pancreatic endocrine cells [13, 41], indicating that the homeodomain of Pax6b is not crucial for endocrine cell differentiation in the pancreas in contrast to eye development. To get a clue whether the mode of action of Pax6b is also similar between EECs and PECs, we next investigated whether the EEC differentiation was affected in the sunrise mutants. FISH revealed no effect of the sunrise mutation on the expression of *pyyb* and of *mlnl* in contrast to the null *pax6b*^*sa0086*^ mutation (Fig. 8c, f). This indicates that the homeodomain of Pax6b is not crucial for the differentiation of endocrine cells both in the intestine and in the pancreas.

## Discussion

By RNA sequencing of FACS-purified cells, we report in this study the transcriptome of enteroendocrine cells (EECs) from zebrafish highlighting the repertoire of peptide hormones and regulatory factors expressed by these cells. The comparison of these RNA-seq data with other zebrafish tissues confirms the close similarity of EECs with pancreatic endocrine cells (PECs). Furthermore, we show that Pax6b is required for the proper expression of many hormones in both the pancreas and intestine, acting via a large set of common gene targets. Inactivation of zebrafish Pax6b does not affect the total number of endocrine cells but affects the balance between endocrine cell subtypes, leading to an increase of ghrelin+/motilin-like+ cells and a reduction of other endocrine cell subtypes in both tissues. The Pax6b homeodomain is also dispensable in the two organs. All these data highlight the high similarity between EECs and PECs.

The present study reveals also that the zebrafish EECs express most of the hormones known to be secreted by mammalian EECs. Only 4 EEC hormones were not detected in zebrafish. Secretin (Sct), pancreatic polypeptide (PP) and gastrin could not be detected as no orthologous gene is present in the zebrafish genome. The absence of *sct* and *pp* genes is in agreement with previous studies indicating the lack of these two genes in teleost [42–44]. The detection of PP by immunofluorescence at the level of zebrafish EECs [29] and zebrafish PECs [45] is probably due to cross-reactions of the antibodies with Pyya and/or Pyyb as significant sequence similarities are observed between Pyy and PP mature peptides (see Additional file 10: Fig. S4). Although the *neurotensin* gene is present in the zebrafish genome, no expression was detected in EECs, at least in 4 dpf larvae. On the other hand, the present transcriptomic profiling of zebrafish EECs reveals the identification of three neuropeptide transcripts not described so far in EECs: *proenkephalin-a* (*penka*), *calcitonin* (*calca*) and *adenylate cyclase-activating polyeptide 1a* (*adcyap1a*). We could confirm at the protein level the expression of two of them, enkephalin and Adcyap1 (Fig. 2). Further studies should be performed to confirm the expression of Calca at the protein level and determine the role of these 3 neuropeptides on the digestive tract. To get a hint whether these hormones could be also expressed in the mammalian intestine, we searched into the NCBI GEO repository and into the Human Protein Atlas database [46] for the presence of these transcripts in the gut. We found that *Penk* is expressed in the colon of 8-week-old male mice [47] and at a low level in the human colon. Similarly, human *CALCA* and more specially *CALCB* (calcitonin-related polypeptide B) transcripts can be detected in bulk RNA-seq of the gastrointestinal tract mainly at the level of the colon. Finally, ADCYAP1 expression is detected in the human gastrointestinal tract mostly in the colon as well as in beta pancreatic cells [48]. Furthermore, the recent single-cell RNA-seq data from the small murine intestine reveal that some enterochromaffin cells express *Adcyap1* at low levels [3]. Thus, all these data suggest that the three hormones Penk, Calca(/b) and Adcyap1 could also be expressed in some mammalian EECs, and their expression should be verified by immunostaining notably at the level of the colon.

Some similarity between EECs and PECs has been previously recognized notably thanks to the shared expression of markers including some hormones and transcription factors. Using the zebrafish, the present study demonstrates the similitude at the transcriptomic level and by identifying a common Pax6b-dependant gene regulatory network. When considering the ontogeny and evolutionary aspects of EECs and PECs, it is tempting to speculate that these two endocrine cell types not only share developmental pathways but also have a common ancestor. Indeed, on the ontogeny aspect, both EECs and PECs derive from the endoderm layer and their specification is controlled by lateral inhibition via the Delta/Notch signalling pathway, leading to the formation of scattered endocrine cells within the epithelium. Inhibition of the Notch pathway allows the activation of proneuronal bHLH factors in endocrine precursors that drive the formation of EECs and PECs [49, 50]. As proposed by others [16], the EECs have probably co-opted for these neuronal gene programmes to trigger their differentiation early during the evolution of metazoa. While the EECs are present in the gut of invertebrates, the first signs of the pancreatic organ appear much later with the first vertebrates: the agnathan species, such as lampreys or hagfishes, possess at the adult stage one or two aggregates of insulin-expressing cells just next to the intestine and near the bile duct without associated exocrine tissue while other endocrine cells expressing glucagon, somatostatin or PYY-like peptides are present within the intestinal epithelium [19, 20, 51]. Furthermore, at the larva stage, some of these insulin+ cells are still detected within the intestinal epithelium of lampreys [52]. Thus, these observations suggest that, when the first vertebrate species appeared, some enteroendocrine cells could move out from the gut to form clusters, possibly through the upregulation of cell adhesion molecules that have led to the formation of pancreatic endocrine islets. This hypothesis is supported by studies on sea urchin larvae showing the presence of cells expressing insulin-like peptide in the gut region located at the stomach-intestine boundary and expressing the pdx1 orthologous gene [18, 53]. Furthermore, the generation of the first PECs in the zebrafish embryos is also consistent with this idea: indeed, the first insulin-expressing cells appear scattered within the anterior endodermal gut epithelium at an early stage (14 hpf) and subsequently migrate dorsally from the embryonic gut to form a cluster and generate the principal pancreatic islet (named the dorsal pancreatic bud) [45]. Thus, all these observations entail the notion that PECs are in fact EEC-like cells, explaining why PECs share so many features with EECs. This concept is also supported by previous reports showing the conversion of EECs into insulin-expressing beta-like cells upon targeted expression or inactivation of some transcription factors [54–56]. The close relatedness between EECs and PECs underlined in our study has also important implications for the interpretation of GWAS for type 2 diabetes (T2D). Indeed, as many of the genomic variants linked to T2D are near (or in) genes expressed in PECs [57, 58], it is widely considered that at least part of the defects causing T2D stems from PEC dysfunctions. However, as the transcriptomes of PECs and EECs are highly similar, the defects causing T2D may also have their origin from EECs.

The present study confirms that Pax6 is required for the proper expression of many hormones from EECs and PECs and changes the proportion of the endocrine cell subtype without affecting the total number of endocrine cells in both the pancreas and intestine. The pancreatic phenotype of zebrafish *pax6b*-null mutants is comparable to the *Pax6* mutant mice as the same major pancreatic hormones are up- and downregulated in the two species, although the effect on beta cells looks more drastic in zebrafish than in mice (Pax6 KO mice displaying only 70% reduction) [11, 59]. The almost complete loss of beta cells in zebrafish may derive from a stronger decrease in the expression of the beta-specific factor Pdx1 (as well as Mnx1 and Nkx6.2) in zebrafish pax6b mutants. On the other hand, in the intestine, there is a striking contrast between zebrafish and mice Pax6 mutants: while Pax6 is required for the expression of several EEC hormones in both species, the identity of these hormones is strikingly divergent. Indeed, amongst the 8 EEC hormones analysed in Pax6 mutant mice, *Gcg* (GLP1 and 2), *Sst* and *Gip* genes were strongly downregulated while *Pyy* was not significantly affected (ghrelin and motilin were not analysed in Pax6 KO murine intestine). In zebrafish, *pax6b* inactivation causes a drastic downregulation of *pyyb* and does not affect significantly *gcga*, *sst2* and *gip* gene expression. The reason for such differences between mice and zebrafish is unclear and probably rely on changes during vertebrate evolution either at the level of genomic Pax6 binding sites or of downstream regulatory programmes. Further studies including ChIP-seq experiments will be required to answer that question.

## Conclusion

By determining the transcriptomic landscape of zebrafish enteroendocrine cells, we report in this study all peptide hormones and regulatory factors expressed in these cells. The close relatedness of EECs and PECs observed at the transcriptomic level was further supported by analysing the effect of pax6b loss-of-function revealing that a large set of genes is regulated similarly by Pax6b in both cell types. These results support the hypothesis of a common phylogenetic origin of EECs and PECs; they also underscore the potential implication of EECs in metabolic diseases such as type 2 diabetes.

## Methods

### Zebrafish maintenance, transgenic and mutant lines and isolation of EECs and PECs by FACS

Zebrafish (*Danio rerio*) were raised according to standard protocols and staged according to Kimmel et al. [60]. The following zebrafish transgenic and mutant lines were used: *Tg(pax6b:GFP)*^*ulg515*^ [61], *TgBAC(pdx1:EGFP)*^*bns13*^ [38], *Tg(− 8.5nkx2.2a:GFP)*^*ia2*^ [62], *pax6b*^*sa0086*^ and *pax6b*^*sunrise*^ [13]. Enteroendocrine cells (EECs) were isolated by dissecting the gut from about 200 *Tg(pax6b:GFP)*^*ulg515*^ larvae at 4 dpf, taking care of not including pancreatic tissue. Cell dissociation was next performed by incubation in HBSS 1× supplemented with 100 U/ml collagenase IV and 0.3 U/ml Dispase (Life Technologies) for 10 min. Cells were washed in HBSS (Mg^2+^ and Ca^2+^ free) containing 1% BSA, and GFP-expressing EECs were selected by two consecutive FACS purifications, the first in the “yield mode” and the second in the “purity mode”, using FACS Aria II. Four replicates of EEC containing about 3000 cells were prepared. Pancreatic endocrine cells (PECs) were also obtained from the *Tg(pax6b:GFP)*^*ulg515*^ line [61] by dissecting the dorsal pancreatic bud from about 200 27-hpf transgenic embryos. FACS selection was performed as described for EECs except that cell dissociation was performed in *Tryple Select* 1X (Gibco) supplemented with 100 U/ml collagenase IV (Life Technologies) for 5 min. For the preparations of EECs and PECs from *pax6b* null mutant embryos, the *pax6*^*sa0086*^ line [13] was first crossed with the *Tg(pax6b:GFP)*^*ulg515*^ line; heterozygous *pax6b*^*sa0086*^ fish harbouring the transgene (pax6b:GFP) were inbred to generate homozygous *pax6b*^*sa0086*^ transgenic embryos which were selected based on the absence or reduction of lens. The isolation of EECs and PECs from *pax6b*^*sa0086*^ homozygotes were performed in triplicates following the same procedure than for the wild-type larvae. The accuracy of *pax6b*^*sa0086*^ homozygote selection was verified after the RNA-seq by checking the presence of the null sa0086 allele in 100% of *pax6b* reads in the mutant samples.

### cDNA synthesis, library preparation and sequencing

Each EEC or PEC sample obtained after FACS was directly pelleted by centrifugation and resuspended in 3.5 μl of *reaction buffer*, lysed by freezing in liquid nitrogen and stored at − 80 °C according to the Smart-seq2 protocol [63]. cDNA was synthesized and amplified by a 13-cycle PCR reaction. The quality of cDNA was verified by 2100 High Sensitivity DNA assay (Agilent Technologies); 1-ng cDNA was used for preparing each cDNA library using the Nextera-XT kit (Illumina) and sequenced on Hi-seq 2000 to obtain around 40–60 millions of reads (100 base paired-ends).

### RNA-seq data analyses

Sequences were trimmed in order to remove adaptors and low-quality bases. Trimmed reads were mapped into the zebrafish genome GRCz11 (Ensembl Release 92, www.ensembl.org) using the STAR software v.2.5.4b [64]. Gene expression was measured from the mapped reads by using built-in STAR module (--quantMode GeneCounts) and are expressed in counts of reads per million (CPM) [65]. The RNA-seq raw data have been deposited in the Gene Expression Omnibus (GEO) under the accession number GSE149081. The comparison of the transcriptome of EECs and PECs with other zebrafish tissues was performed using the DESeq2 R package [66]. This comparison includes RNA-seq data from the heart (ArrayExpress: E-MTAB-460; GEO: GSE71755), brain (ArrayExpress: E-MTAB-460), pancreatic cells [24, 67], liver (GEO: GSE82246) and intestine (GEO: GSE83195). The reproducibility of RNA-seq data from wild-type and *pax6b* mutants was verified by a principal component analysis (PCA) obtained using the DESeq R package [68]. Differential expression (DE) analysis was performed using the R package DESeq2 [66], to identify all genes displaying significant change in the expression between wild-type and *pax6b* mutants (with fold discovery rate < 10%). The significance in the overlap of the sets of genes regulated by Pax6b in EECs and in PECs was determined with the R built-in statistical Fisher’s exact test.

### Gene Ontology enrichment analysis

Gene Ontology (GO) enrichment analysis was performed on the different gene sets (genes selectively expressed either in EECs or in PECs, pax6b-regulated genes only in PECs, only in EECs and in both PECs and EECs) using the DAVID bioinformatics resources 6.8 [69] taking as background all the zebrafish genes. The enrichment analysis was focused on the GO biological process, molecular function and KEGG pathways with a statistical Fisher exact test *p* value < 0.05.

### In situ hybridization and immunohistochemistry

Antisense RNA probe for the different genes was prepared as described by Thisse and Thisse [70]. Briefly, primers were designed to amplify a part of the transcript that is used as a template to synthesize the probe. The reverse primer at the 5′ contains the minimal promoter sequence for T3 RNA polymerase (5′-AATTAACCCTCACTAAAGGGAG-3′); templates were amplified by RT-PCR. Whole-mount in situ hybridization and fluorescent in situ hybridization (WISH and FISH, respectively) were performed as described previously [71] on wild-type (AB strain), pax6sa0086 null mutant or pax6bsunrise mutant embryos/larvae [13]. Immunohistochemistry (IHC) on whole-mount embryos/larvae was performed as described [72]. The antibodies used are the guinea pig polyclonal anti-PDX1 antibody (ab47308, Abcam) used at a dilution of 200×, the chicken anti-GFP (Aves lab) used at a dilution of 1000×, the rabbit anti-enkephalin (T4294; Peninsula Laboratories International, Inc.) used at a 400× dilution, the rabbit anti-PACAP (Adcyap1) (T-4473.0050; from Peninsula Laboratories International, Inc.) used at a 300× dilution, the monoclonal mouse Hu antibody (16A11: Invitrogen Cat. #A-21271) used at a 1000× dilution and Alexa Fluor secondary antibodies at 1000× dilution. Stained embryos were mounted in Prolong (Invitrogen) with DAPI and imaged using SP5 confocal microsope (Leica). For the immunostainings on adult tissue, the intestine of transgenic *Tg(-8.5nkx2.2a:GFP)*^*ia2*^ adult zebrafish was dissected and fixed for 1 day in PFA 4% rinsed in PBS and embedded in TEK using standard procedures and sectioned using cryostat for generating 10-μm sections which were mounted on glass slides. The primary and secondary antibodies were used as the same dilution as described above for whole-mount larvae.

## Supplementary information

**Additional file 1: Figure S1.** The pax6b:GFP transgene is not expressed in enteric neurones. Immunofluorescence on 5 dpf *Tg(pax6b:GFP)* larva using antibodies against GFP (green) and against the enteric neurone marker Hu (red). Confocal vues of the bulb intestine (upper panels), mid-intestine (middle panels) and posterior intestine (lower panels) showing no colocalisation of GFP with Hu. Dapi staining is in blue. Scale bar =50μm.

**Additional file 2: Table S1.** Expression level of all genes in zebrafish enteroendocrine cells. The expression levels is given for all genes in CPM (counts per million) for the four EEC samples (column C to F) as well as the mean and the standard deviation obtained for each gene (columns G and H). The last column gives the mean expression levels in FPMK.

**Additional file 3: Figure S2.** Quantification of EECs expressing different neuropeptide transcripts in 4 dpf zebrafish larvae. The number of EECs expressing each hormones was determined by counting the labelled cells after WISH using the corresponding hormone probes on 4 dpf larvae. Each point in the graph represents the number of labelled cells in one larva. Bars represent the mean values and S.E.

**Additional file 4: Figure S3.** Expression of *vipb* and *adcyap1a* transcripts in zebrafish EECs. Confocal images of the zebrafish gut from larvae stained by double fluorescent in situ hybridization (FISH) using the enteric neurone marker *phox2b* (green) and the *vipb* or *adcyap1a* probes (red). (A) general vue of the gut showing co-localisation of *vipb* and *phox2b* in ENs at the level of anterior intestine (upper left part of the image) and *vipb+* EECs in the posterior intestine (bottom right of image). (B) higher magnification of the gut showing three vipb+ phox2b+ ENs and two vipb+ EECs. (C) adcyap1a+ cells are distinct from phox2b+ ENs which are located outside the intestinal epithelium (Dapi staining in blue).

**Additional file 5: Table S2.** List of transcription factors expressed in both EECs and PECs, specific for EECs and specific for PECs. The expression level is given in FPKM. The TF expressed in both PECs and EECs were selected based on their expression level above the threshold of 1 FPKM in both cell types. The TF expressed specifically in PECs were obtained by selecting those expressed above 1 FPKM only in PECs and with an expression ratio of PEC/EEC above 5-fold. Inversely, EEC-specific TF were obtained by selecting those expressed above 1 FPKM only in EECs and with an expression ration EEC/PEC above 5-fold.

**Additional file 6: Table S3.** Lists of genes selectively expressed in EECs and in PECs with their gene ontology enrichment analyses. The genes specifically expressed in PECs were selected by their expression above 5 CPM in PECs and below 1 CPM in EECs (sheet1). Inversely EEC-specific genes were selected by their expression above 5 CPM in EECs and below 1 in PECs (sheet 2). The GO terms obtained for the PEC-specific genes and the EEC-specific genes are given in sheets 3 and 4 respectively.

**Additional file 7: Table S4.** Expression level of all genes EECs and PECs (wild type and pax6b^sa0086^). The expression level of all genes is given for wild-type and *pax6b* mutant EECs and PECs in normalized CPM. The expression levels in each sample are given in the excel sheet 1(“CPM samples”) and the means and standard deviations are given in excel sheet 2 (“mean and Sdt dev.”).

**Additional file 8: Table S5.** List of Pax6b-regulated genes and GO enrichment analysis. The excel sheets 1, 3 and 5 give respectively the lists of genes regulated by Pax6b in both PECs and EECs (sheet 1), in PECs only (sheet 3) and in EECs only (sheet 5) together with the expression level in normalized CPM in wild type and pax6b mutant EECs and PECs, the expression fold changes and the P-adjusted values. The excel sheets 2, 4 and 6 display respectively the enrichment of GO terms and pathways for the Pax6b-regulated genes in PECs and EECs (sheet 2), in PECs only (sheet 4) and in EECs only (sheet 6) together with the *P*-values for the Fischer exact test and the lists of genes associated to each GO term.

**Additional file 9: Table S6.** list of markers for each pancreatic endocrine cell subtypes. The list of markers for the pancreatic alpha-, beta-, delta- and epsilon- cell subtypes was obtained from previous published RNA-seq studies [24, 40]. This list was used to determine the proportion of cell subtype markers regulated by Pax6b as shown in the upper panel of Fig. 7.

**Additional file 10: Figure S4.** Sequence similarity between pancreatic polypeptide and of peptide YY. Alignment of the amino-acid sequence of pancreatic polypeptide (PP) from mice and rat with peptide YY from mice and zebrafish (DrPyy A or b).

## Data Availability

All data generated or analysed during this study are included in this published article, its supplementary information files and publicly available repositories. The raw RNA-seq datasets of EECs and PECs from wild-type and *pax6b* mutants have been deposited on Gene Expression Omnibus (GEO) under the accession number GSE149081 (https://www.ncbi.nlm.nih.gov/geo/query/acc.cgi?acc=GSE149081). The EEC and PEC RNA-seq were compared to other published RNA-seq data from the heart (ArrayExpress: E-MTAB-460; GEO: GSE71755), brain (ArrayExpress: E-MTAB-460), liver (GEO: GSE82246) and intestine (GEO: GSE83195).

## Abbreviations

EEC: Enteroendocrine cell
PEC: Pancreatic endocrine cell
EN: Enteric neuron
WISH: Whole-mount in situ hybridization
FISH: Fluorescent in situ hybridization
CPM: Count of reads per million
FPKM: Fragments per kilobase of exon model per million reads mapped
S.E: Standard errors
Gcg: Glucagon
Sst: Somatostatin
Ghrl: Ghrelin
Pyy: Peptide YY
Ckk: Cholecystokinin
Gip: Glucose-dependent insulinotropic polypeptide
Vip: Vasoactive intestinal peptide
mlnl: Motilin-like
Insl5: Insulin-like peptide 5
Nmba: Neuromedin B a
Adcyap1: Adenylate cyclase-activating polypeptide 1
Galn: Galanin
Calca: Calcitonin
Penka: Proenkephalin-a
