# Supplementary figures and images for: Pancreatic and intestinal endocrine cells in zebrafish share common transcriptomic signatures and regulatory programmes
Source: BMC Biol. 2020 Aug 31;18:109. doi: 10.1186/s12915-020-00840-1 (PMC7457809; doi:10.1186/s12915-020-00840-1)

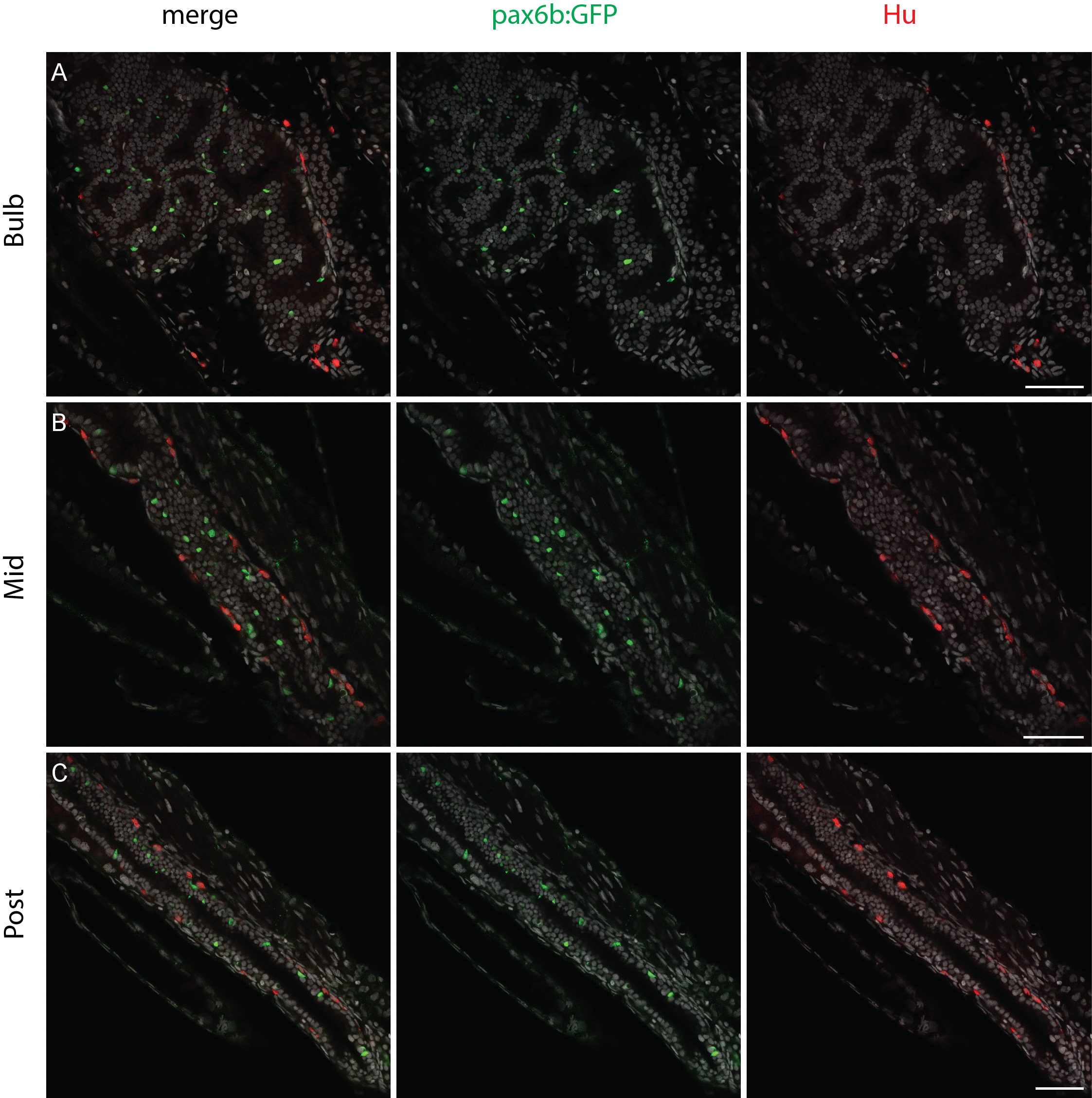

Supplement: Supplementary file 1 — Additional file 1: Figure S1. The pax6b:GFP transgene is not expressed in enteric neurones. Immunofluorescence on 5 dpf Tg(pax6b:GFP) larva using antibodies against GFP (green) and against the enteric neurone marker Hu (red). Confocal vues of the bulb intestine (upper panels), mid-intestine (middle panels) and posterior intestine (lower panels) showing no colocalisation of GFP with Hu. Dapi staining is in blue. Scale bar =50μm. [file 12915_2020_840_MOESM1_ESM.png]

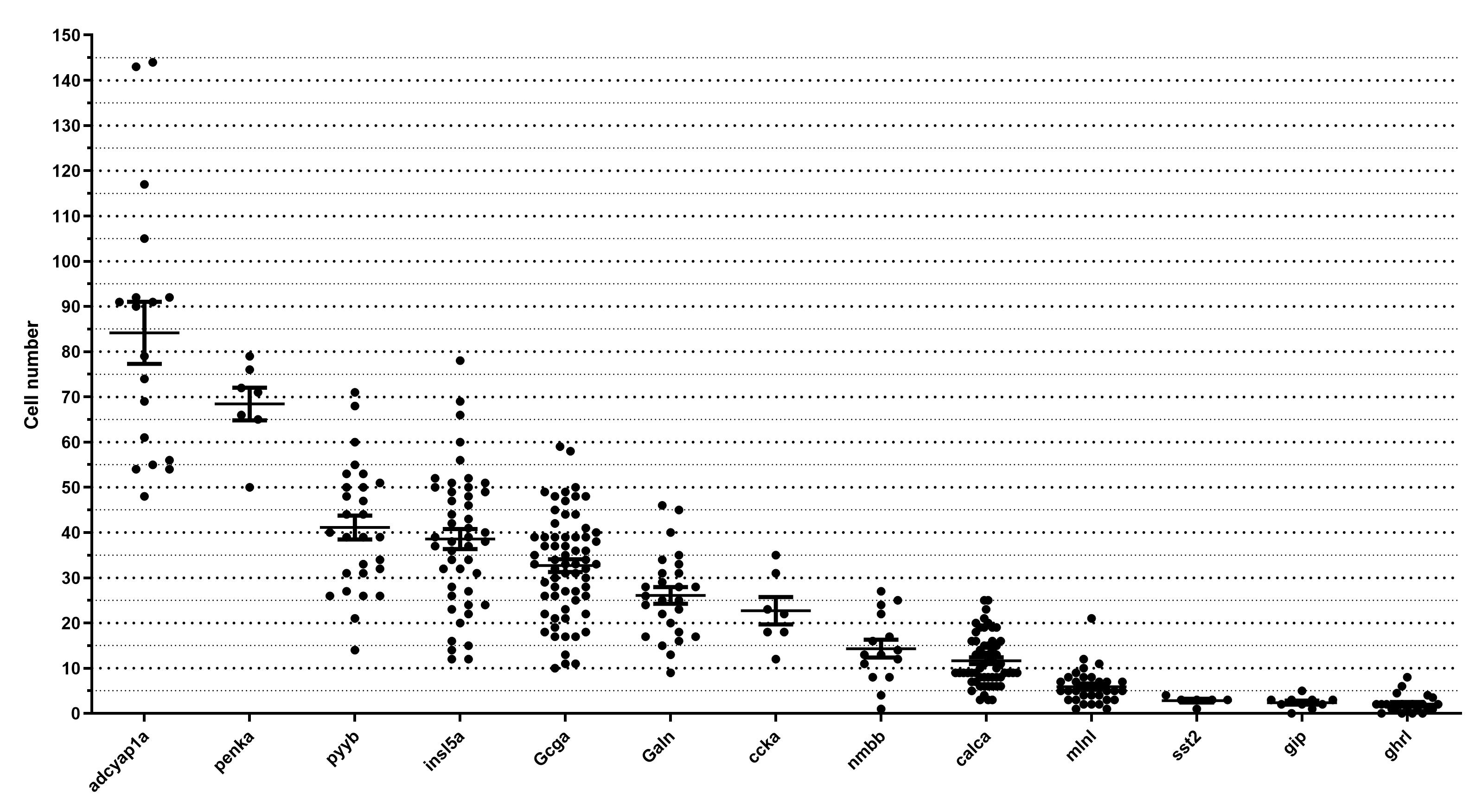

Supplement: Supplementary file 3 — Additional file 3: Figure S2. Quantification of EECs expressing different neuropeptide transcripts in 4 dpf zebrafish larvae. The number of EECs expressing each hormones was determined by counting the labelled cells after WISH using the corresponding hormone probes on 4 dpf larvae. Each point in the graph represents the number of labelled cells in one larva. Bars represent the mean values and S.E. [file 12915_2020_840_MOESM3_ESM.png]

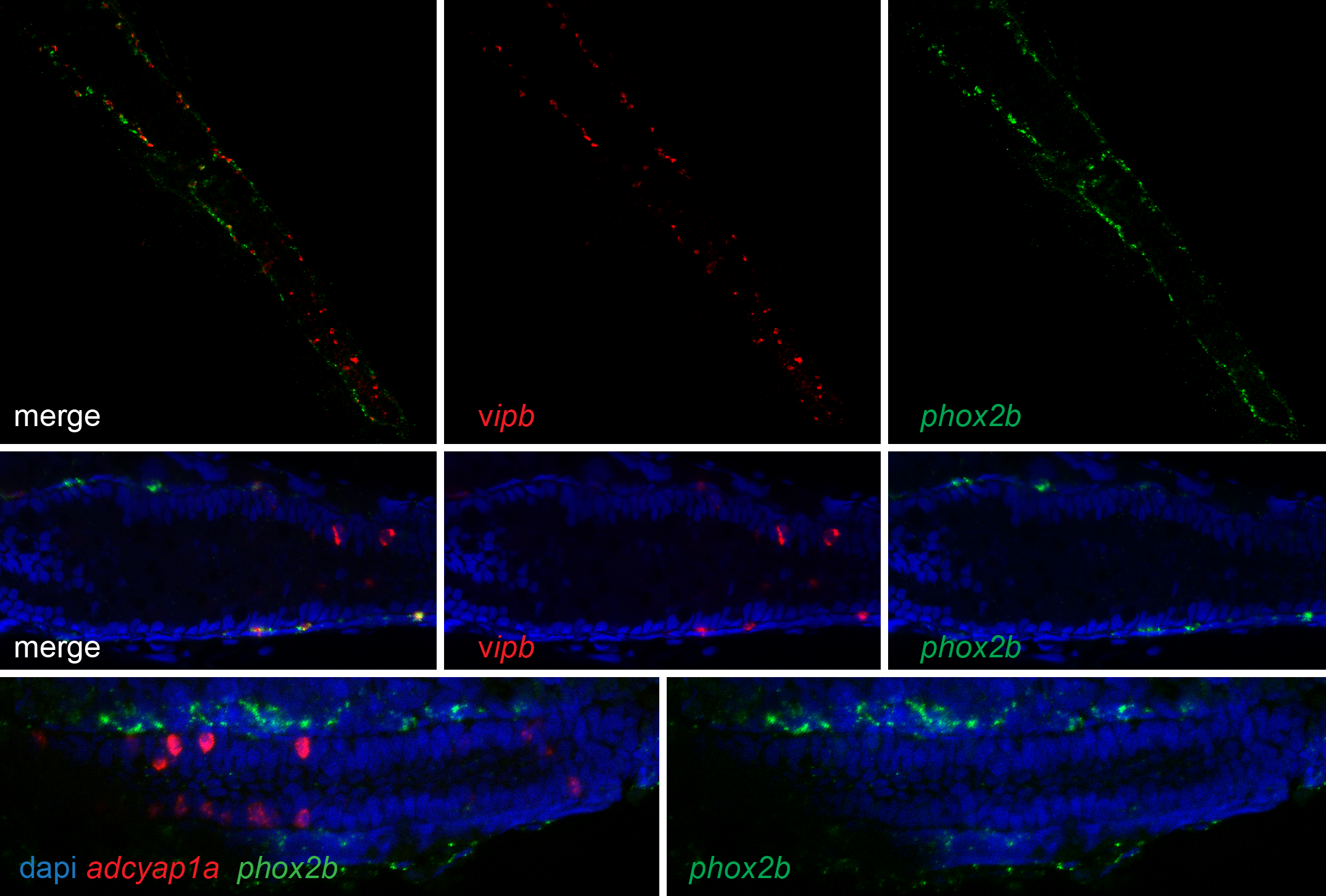

Supplement: Supplementary file 4 — Additional file 4: Figure S3. Expression of vipb and adcyap1a transcripts in zebrafish EECs. Confocal images of the zebrafish gut from larvae stained by double fluorescent in situ hybridization (FISH) using the enteric neurone marker phox2b (green) and the vipb or adcyap1a probes (red). (A) general vue of the gut showing co-localisation of vipb and phox2b in ENs at the level of anterior intestine (upper left part of the image) and vipb+ EECs in the posterior intestine (bottom right of image). (B) higher magnification of the gut showing three vipb+ phox2b+ ENs and two vipb+ EECs. (C) adcyap1a+ cells are distinct from phox2b+ ENs which are located outside the intestinal epithelium (Dapi staining in blue). [file 12915_2020_840_MOESM4_ESM.png]

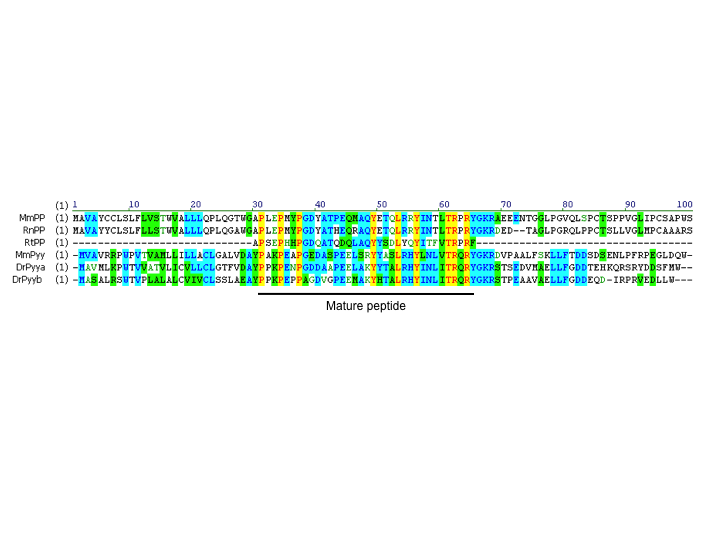

Supplement: Supplementary file 10 — Additional file 10: Figure S4. Sequence similarity between pancreatic polypeptide and of peptide YY. Alignment of the amino-acid sequence of pancreatic polypeptide (PP) from mice and rat with peptide YY from mice and zebrafish (DrPyy A or b). [file 12915_2020_840_MOESM10_ESM.png]
